# The mixed-lineage kinase 3 inhibitor URMC-099 facilitates microglial amyloid-β degradation

**DOI:** 10.1186/s12974-016-0646-z

**Published:** 2016-07-11

**Authors:** Weiguo Dong, Christine M. Embury, Yaman Lu, Sarah M. Whitmire, Bhagyalaxmi Dyavarshetty, Harris A. Gelbard, Howard E. Gendelman, Tomomi Kiyota

**Affiliations:** Department of Pharmacology and Experimental Neuroscience, University of Nebraska Medical Center, Omaha, NE 68198-5930 USA; Department of Integrated Traditional Chinese and Western Medicine, Fujian University of Traditional Chinese Medicine, Fuzhou, Fujian 350122 People’s Republic of China; Department of Neurology, Center for Neural Development & Disease, School of Medicine and Dentistry, University of Rochester Medical Center, Rochester, 14642 NY USA; Department of Internal Medicine, University of Nebraska Medical Center, Omaha, 68198-5880 NE USA

**Keywords:** Mixed-lineage kinase 3, Alzheimer’s disease, Amyloid-β, Microglia, Phagocytosis, Endolysosomal pathway

## Abstract

**Background:**

Amyloid-β (Aβ)-stimulated microglial inflammatory responses engage mitogen-activated protein kinase (MAPK) pathways in Alzheimer’s disease (AD). Mixed-lineage kinases (MLKs) regulate upstream MAPK signaling that include p38 MAPK and c-Jun amino-terminal kinase (JNK). However, whether MLK-MAPK pathways affect Aβ-mediated neuroinflammation is unknown. To this end, we investigated if URMC-099, a brain-penetrant small-molecule MLK type 3 inhibitor, can modulate Aβ trafficking and processing required for generating AD-associated microglial inflammatory responses.

**Methods:**

Aβ1-42 (Aβ42) and/or URMC-099-treated murine microglia were investigated for phosphorylated mitogen-activated protein kinase kinase (MKK)3, MKK4 (p-MKK3, p-MKK4), p38 (p-p38), and JNK (p-JNK). These pathways were studied in tandem with the expression of the pro-inflammatory cytokines interleukin (IL)-1β, IL-6, and tumor necrosis factor (TNF)-α. Gene expression of the anti-inflammatory cytokines, *IL-4* and *IL-13*, was evaluated by real-time quantitative polymerase chain reaction. Aβ uptake and expression of scavenger receptors were measured. Protein trafficking was assessed by measures of endolysosomal markers using confocal microscopy.

**Results:**

Aβ42-mediated microglial activation pathways were shown by phosphorylation of MKK3, MKK4, p38, and JNK and by expression of IL-1β, IL-6, and TNF-α. URMC-099 modulated microglial inflammatory responses with induction of *IL-4* and *IL-13*. Phagocytosis of Aβ42 was facilitated by URMC-099 with up-regulation of scavenger receptors. Co-localization of Aβ and endolysosomal markers associated with enhanced Aβ42 degradation was observed.

**Conclusions:**

URMC-099 reduced microglial inflammatory responses and facilitated phagolysosomal trafficking with associated Aβ degradation. These data demonstrate a new immunomodulatory role for URMC-099 to inhibit MLK and to induce microglial anti-inflammatory responses. Thus, URMC-099 may be developed further as a novel disease-modifying AD therapy.

**Electronic supplementary material:**

The online version of this article (doi:10.1186/s12974-016-0646-z) contains supplementary material, which is available to authorized users.

## Background

Neuroinflammation is a pathogenic driver for Alzheimer’s disease (AD) [1–3]. Amyloid-β (Aβ)-activated microglia secrete pro-inflammatory neurotoxins that are strongly linked to AD-associated neural injury. Such inflammatory responses are notably tied beyond AD to common age-related neurodegenerative, neuroinfectious, and neuroinflammatory disorders [4–13]. Activated microglia, in turn, stimulate neurons to produce more Aβ and the microtubule-associated protein tau, in a vicious paracrine loop [14]. The events change the brain’s microenvironment and further affect microglial activation, leading to progressive neuronal injuries. The end result is a paracrine feedback of neurotoxin amplification that drives disease with Aβ serving as the principal inducer of innate immune activation [15–17]. This has led many researchers to develop the means to harness immunity for therapeutic gain. Notably, Aβ vaccination can effectively clear Aβ in both mouse models of human disease and AD patients [18, 19]. For treatment of AD, Aβ immunization reduces the Aβ42 load with immunomodulation, including microglial phagocytosis and changed lysosomal and scavenger markers [20, 21]. However, such immunizations can also result in the development of meningoencephalitis (~6 %) [22], spongiosis, and neuronal loss [23]. Since removal of plaques at later stages of disease marked by neurofibrillary tangle formation was not proven to be beneficial, alternative treatment strategies are likely required. These might improve clinical outcomes if administered years before clinical signs and symptoms emerge. Indeed, such a therapeutic approach could speed clearance of Aβ and modify inflammatory activities before disease develops.

Prior therapeutic approaches focused on development of immunomodulatory agents that affect AD microglial responses. For example, nonsteroidal anti-inflammatory drugs (NSAIDs) or antioxidants reduce harmful microglial inflammatory activities and protect neurons in animal models of human disease [24, 25]. While the drugs are effective in converting microglial polarization from an M1 (classical activation) to an M2 (alternative activation) phenotype and attenuating neurotoxin production, successful human therapeutic translation remains out of reach [1, 25, 26].

While the “selectively non-selective” brain-penetrant mixed-lineage kinase type 3 (MLK3) inhibitor, URMC-099, can attenuate neuroinflammatory responses and facilitate the actions of long-acting nanoformulated antiretroviral drugs during human immunodeficiency virus-1 (HIV-1) infection [27–29], it is not known whether it could also harness autophagy. If operative, this could occur as a secondary response to phagocytosis of Aβ. Here, we show yet another novel therapeutic role of URMC-099 as an immune modulator in microglial inflammation and phagocytosis through accelerating phagolysosomal pathway Aβ degradation. In this manner, we posit that the drug could be developed as a novel AD therapeutic candidate.

## Methods

### Microglia isolation and cultivation

Housing, care, and breeding of non-transgenic mice (B6.129 hybrid background) were approved by the Institutional Animal Care and Use Committee of the University of Nebraska Medical Center. Primary cultured mouse microglia were prepared from the postnatal day 1 newborn mouse brains [30–32]. Meninges-free newborn mouse cortices were minced and trypsinized, followed by mechanical dissociation and filtration to remove tissue chunks. Cells were plated onto plastic tissue culture bottles as mixed glial cultures in Dulbecco’s modified eagle medium supplemented with heat-inactivated 10 % fetal bovine serum, 50 μg/ml penicillin/streptomycin (Life Technologies, Carlsbad, CA, USA) and macrophage colony-stimulating factor (MCSF). Microglia were released from astrocytes in the tissue culture media by shaking. Non-adherent cells were collected 7–14 days after plating.

### Aβ activation and URMC-099 treatment

Microglia were plated at a density of 2 × 10^5^ onto 24-well plates for RNA/protein extraction or onto 24-well plates with glass coverslips for confocal microscopy. 2 × 10^4^ cells were placed in each 96-well plate for immunofluorescence studies. One day before Aβ exposure and URMC-099 treatment, media was replaced without MCSF. Microglia were pre-incubated for 30 min with URMC-099 (100 nM) [28]. Notably, URMC-099 administered at 100 nM did not affect microglial viability as measured by the 3-(4,5-dimethylthiazol-2-yl)-2,5-diphenyltetrazolium bromide (MTT) assay (Additional file 1: Figure S1) that was performed as previously described [33]. After pre-incubation with URMC-099, microglia were incubated with media containing the monomeric Aβ42 (10 μM, Life Technologies, Carlsbad, CA, USA) prepared as previously described [32]. Since monomeric rather than oligomeric Aβ1-42 (Aβ42) peptides are effectively phagocytosed by human monocyte-derived macrophages [34], monomeric Aβ42 was used. After Aβ treatment, microglia were harvested for total RNA or protein extraction or fixed with freshly depolymerized 4 % paraformaldehyde (PFA) in PBS for 15 min for immunofluorescence assays.

### Immunoblot and ELISA tests

Microglial cells were harvested using ice-cold RIPA buffer with protease and phosphatase inhibitor cocktail at end points and centrifuged at 15,000*x*g for 10 min at 4 °C to collect cell lysates. Protein concentrations were determined using a Micro BCA Protein Assay (Thermo Fisher Scientific, Waltham, MA, USA). For immunoblots, protein lysates were diluted 1:1 with Laemmli buffer containing β-mercaptoethanol, incubated at 100 °C for 5 min, electrophoresed on sodium dodecyl sulfate-polyacrylamide gels, and electroblotted to 0.45-μm pore size polyvinylidene fluoride membranes (Immobilon-P, Millipore, Billerica, MA, USA). Membranes were blocked in 3 % bovine serum albumin/TBST or 5 % skim milk/TBST and incubated with antibodies (Abs) to phospho-MKK3 (p-MKK3), total MKK3, phospho-MKK4 (p-MKK4), total MKK4, phospho-p38 (p-p38), total p38, phosphor-JNK (p-JNK), total JNK (1:1000, Cell Signaling Technology, Danvers, MA), CD36, CD47 (1:200, Santa Cruz Biotechnology, Santa Cruz, CA, USA), and Aβ (6E10, 1:1000, Covance, Emeryville, CA, USA), at 4 °C for overnight, followed by 30-min incubation in 3 % bovine serum albumin/TBST or 5 % skim milk/TBST with HRP-conjugated anti-goat, mouse, or rabbit IgG Abs (1:2000, Santa Cruz Biotechnology, Santa Cruz, CA, USA). Immunoreactive bands were detected with SuperSignal West Pico or Femto Chemiluminescent substrate and captured using a myECL Imager (Thermo Fisher Scientific, Waltham, MA, USA). After detection of the bands, membranes were incubated with Restore Western Blot Stripping Buffer (Thermo Fisher Scientific, Waltham, MA, USA) and were then used to detect β-actin for normalization using HRP-conjugated anti-β-actin monoclonal (Sigma, St. Louis, MO, USA). For quantitative analysis, ImageJ software (NIH, Bethesda, MD, USA) was used to quantify band intensities relative to total proteins or control β-actin expression. For ELISA, tissue culture media were subjected to capture antibodies for IL-1β, IL-6, and TNF-α (PeproTech, Rocky Hill, NJ, USA) according to the manufacturer’s instructions. For Aβ42 ELISA, cell lysates and tissue culture media were subjected to Aβ42 ELISA (Life Technologies, Carlsbad, CA, USA) according to the manufacturer’s instructions.

### Immunofluorescence and confocal microscopy

Immunofluorescence was performed using pan-Aβ Ab (rabbit polyclonal, 1:100) and Alexa Fluor®488-conjugated anti-rabbit IgG (1:1000), followed by 30 min counterstaining with 4′,6-diamino-2-phenyl-indole (DAPI) (all from Life Technologies, Carlsbad, CA, USA). Images were captured using DP Controller and DP Manager with a DP71 digital camera (Olympus, Orangeburg, NY, USA) attached to a Nikon Eclipse TE300 inverted microscope (Nikon, Melville, NY, USA). For confocal microscopy, PFA-fixed microglia were immunostained with Abs to Aβ (6E10, 1:1000, Covance, Emeryville, CA, USA), Rab5, Rab7 (rabbit polyclonal, 1:500, Santa Cruz Biotechnology, Santa Cruz, CA, USA), and Lamp1 (rabbit polyclonal, ab24170, 1:500, Abcam, Cambridge, MA, USA). Alexa Fluor 488 goat anti-mouse IgG and Alexa Fluor 568 goat anti-rabbit IgG were used as secondary, followed by 30-min counterstaining with DAPI (Life Technologies, Carlsbad, CA, USA). Images were captured using a 63X oil lens on a LSM 710 confocal microscope (Carl Zeiss Microimaging Inc., Thornwood, NY, USA). Images were quantified using ImageJ software with a co-localization plugin (https://imagej.nih.gov/ij/plugins/colocalization.html) (NIH, Bethesda, MD, USA).

### RNA extraction and transcript analyses

Total RNA was extracted from microglia using TRIzol (Life Technologies, Carlsbad, CA, USA). For PCR-based gene expression analyses, cDNA was synthesized with 1 μg of total RNA as a template using a Verso cDNA synthesis kit (Thermo Fisher Scientific, Waltham, MA, USA), and quantitative real-time RT-PCR (RT2-qPCR) was performed on a thermocycler (Mastercycler Gradient, Eppendorf Scientific Inc., Westbury, NY, USA) using 2x SYBR Green qPCR Master Mix (Biotool.com, Houston, TX, USA) and gene specific primer sets (Table 1). All primer sequences were obtained from PrimerBank (https://pga.mgh.harvard.edu/primerbank/) [35]. Thermal cycler conditions were as follows: 10 min at 95 °C for activation of polymerase, followed by 40 cycles of a two-step PCR (95 °C for 15 s and 60 °C for 1 min). Relative expression for target genes was determined by the ΔΔCt method and normalized with glyceraldehyde-3-phosphate dehydrogenase (*Gapdh*) gene expression as an internal control. Each ΔCt value was determined by subtracting *Gapdh* Ct value from the target gene Ct value. The ΔΔCt was calculated by subtracting the ΔCt value of the control from the ΔCt value of other groups. 2^−ΔΔCt^ represented the average relative amount of mRNA to control for each target gene.

**Table 1:** Primers for qPCR analysis

Genes	Forward (5′–3′)	Reverse (5′–3′)
IL-1β	GTGTCTTTCCCGTGGACCTTC	CGGAGCCTGTAGTGCAGTTG
IL-6	TAGTCCTTCCTACCCCAATTTCC	TTGGTCCTTAGCCACTCCTTC
TNF-α	ACTCCAGGCGGTGCCTATG	CCCTGCCACAAGCAGGAAT
IL-4	GGTCTCAACCCCCAGCTAGT	GCCGATGATCTCTCTCAAGTGAT
IL-13	CCTGGCTCTTGCTTGCCTT	GGTCTTGTGTGATGTTGCTCA

### Statistical analyses

All data were normally distributed and presented as mean values ± standard errors of the mean (SEM). In the case of single mean comparison, data were analyzed by Student’s *t* test. In case of multiple mean comparisons, the data were analyzed by one-way ANOVA. When there were significant differences between ≥3 sample means, post hoc comparisons with the Newman–Keuls method was performed using statistics software (Prism 4.0, Graphpad Software, San Diego, CA, USA). A value of *p* < 0.05 was regarded as a significant difference.

## Results

### URMC-099 inhibits microglial p38 and JNK MAPK signaling cascades

**Fig. 1:**
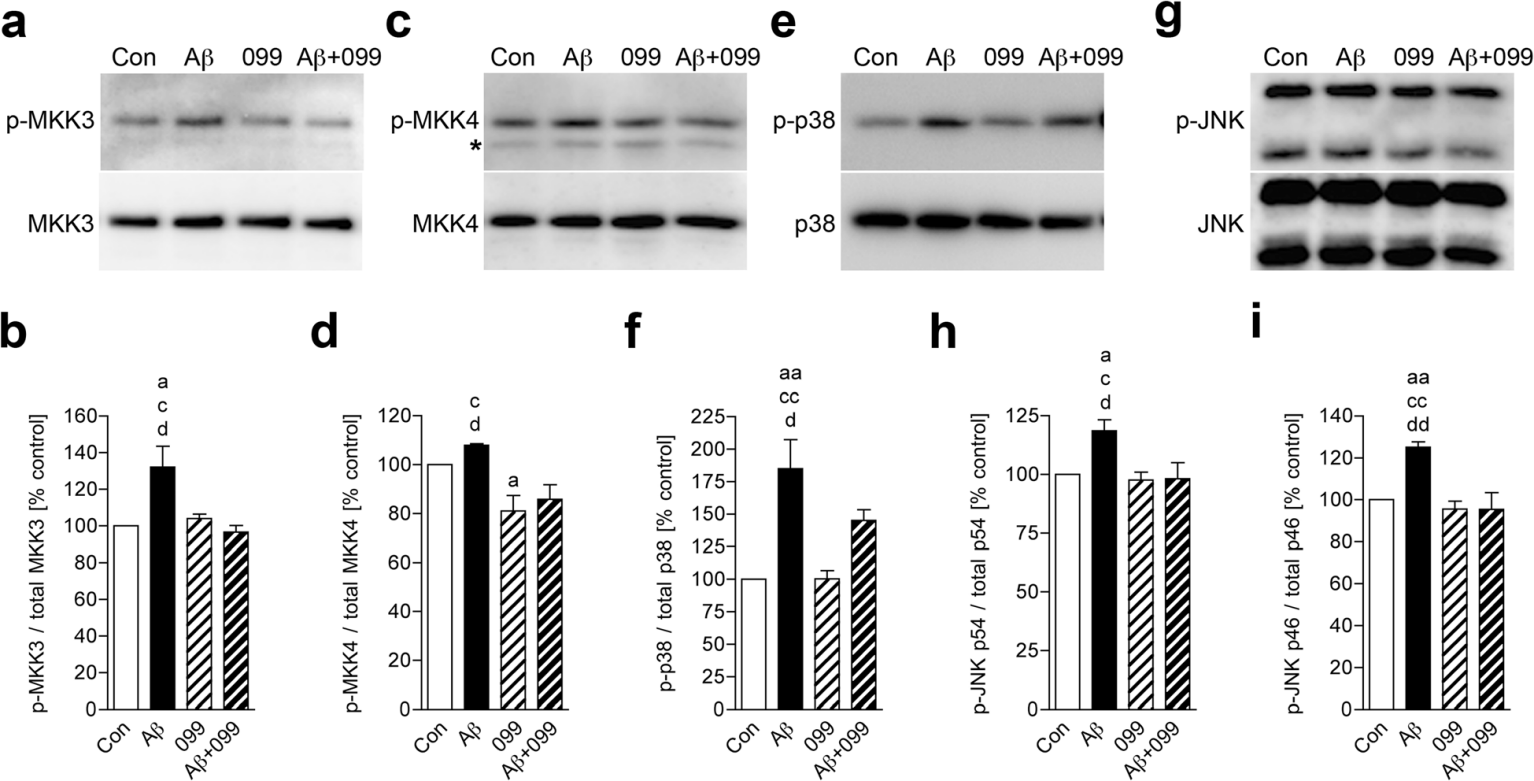
URMC-099 inhibits p38 and JNK MAPK signaling cascades in Aβ-stimulated microglia. Immunoblots of p-MKK3 and total MKK3 (**a**), p-MKK4 (*asterisk*; non-specific bands) and total MKK4 (**c**), p-p38 and total p38 (**e**), p-JNK p54 (*top*), p46 (*bottom*), and total JNK (**g**). Quantification of p-MKK3 (**b**), p-MKK4 (**d**) p-p38 (**f**), p-JNK p54 (**h**), and p46 (**i**) levels. Data are presented as mean ± SEM, ^a,c,d^ *p* < 0.05, ^aa,cc,dd^ *p* < 0.01, ^a^ vs control, ^c^vs URMC-099, ^d^vs Aβ + URMC-099, one-way ANOVA, Newman–Keuls post hoc test

Aβ activates p38 and JNK MAPK cascades to produce pro-inflammatory mediators in microglia [36–38], and has previously been shown to activate MLK3 in cortical neurons [39], but not microglia. Thus, we tested whether URMC-099 could affect the MAPK cascades in Aβ42-stimulated microglia (Fig. 1). As MKK3/6 and MKK4/7 regulate p38 and JNK phosphorylation downstream MLK3 [40], we examined the phosphorylation patterns of MKK3 and MKK4 (Fig. 1a, c). A 30-min Aβ42 treatment increased MKK3 phosphorylation 32.1 % as compared to untreated controls (Fig. 1b). Moreover, trends in MKK4 phosphorylation were also seen with an 8.0 % increase when compared to untreated controls (Fig. 1d). However, URMC-099 treatment significantly reduced both MKK3 and MKK4 phosphorylation in the Aβ-stimulated microglia with decreases of 26.9 and 20.5 % in MKK3 and MKK4 phosphorylation, respectively (Fig. 1b, d). The phosphorylation of p38 and p46/p54-JNK downstream MKK3 and MKK4 was also assayed (Fig. 1e, g). While a 30-min exposure to Aβ42 showed a rapid induction of both p38 and p46/p54-JNK phosphorylation (increases of 84.8, 18.7, and 25.1 % in p38, p46-JNK, and p54-JNK, respectively, as compared to untreated controls, Fig. 1f, h, i), URMC-099 treatment resulted in significant reduction in p38 and JNK phosphorylation in the Aβ-stimulated microglia. Here, decreases were seen at 21.5, 17.3, and 23.7 % in p38, p46-JNK, and p54-JNK, respectively (Fig. 1f, h, i). To assess the effects of URMC-099 on Aβ42-mediated microglial cytotoxicity, we measured cell viability using the MTT assay. Aβ42-treatment reduced cell viability (Additional file 2: Figure S2) as observed previously [31]. Although URMC-099 treatment did not ameliorate Aβ42-mediated cytotoxicity, trends in protection of cell viability were observed with 9.4 and 16.0 % increase in 3- and 48-h incubation, respectively, when compared to Aβ42 treatment (Additional file 2: Figure S2). These results support the notion that URMC-099 inhibits MLK3-MKK3/4-mediated activation of p38 and JNK MAPK cascades in Aβ-activated microglia.

### URMC-099 inhibits activated microglial pro-inflammatory cytokines

**Fig. 2:**
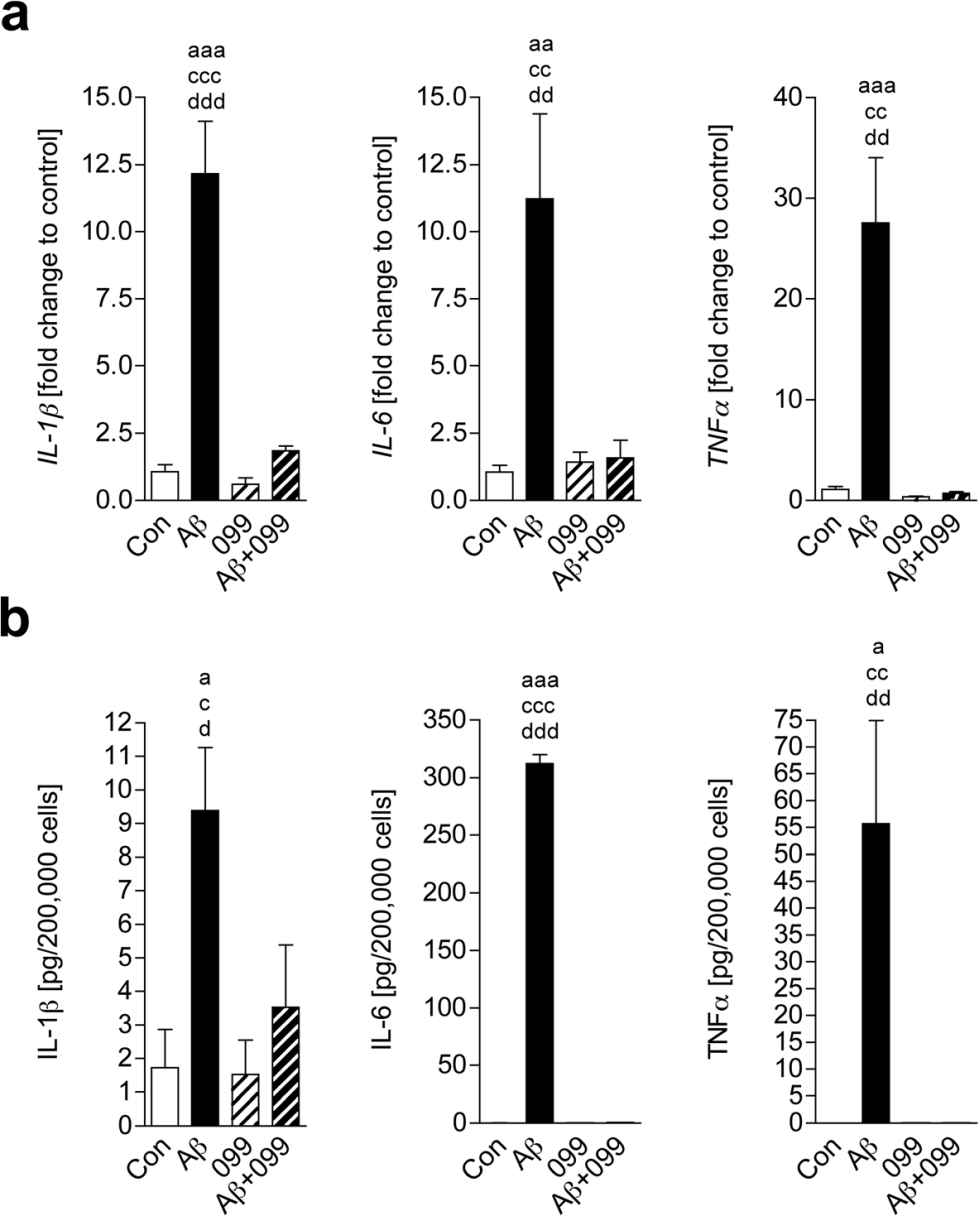
URMC-099 inhibits Aβ-activated microglial pro-inflammatory cytokines. **a** A conventional RT2-qPCR was performed to measure IL-1β, IL-6, or TNF-α expression using primer sets (Table 1) and synthesized cDNA with total RNA isolated from murine microglia (*n* = 3 per group). **b** Quantification of IL-1β, IL-6, or TNF-α protein secretion from murine microglia (*n* = 3 per group). Data are presented as mean ± SEM, ^a,c,d^ *p* < 0.05, ^aa,cc,dd^ *p* < 0.01, ^aaa^ *p* < 0.001, ^a^vs control, ^c^vs URMC-099, ^d^vs Aβ + URMC-099, one-way ANOVA, Newman–Keuls post hoc test

URMC-099 exerts anti-inflammatory effects on microglia exposed to the HIV-1 Tat [28]. To determine whether URMC-099 has similar effects for β-amyloidosis, cultured murine microglia were treated with URMC-099 and exposed to soluble Aβ42 for 30 min. While Aβ42 stimulation induced IL-1β, IL-6, and TNF-α gene expression, URMC-099 reversed this induction (decreases of 84.8, 85.9, and 97.4 % to Aβ42 stimulation for IL-1β, IL-6, and TNF-α, respectively, Fig. 2a). The qPCR results were validated by measuring protein production of pro-inflammatory cytokines in media. After 48-h Aβ42 stimulation, URMC-099 treatment inhibited IL-1β, IL-6, and TNF-α (decreases of 62.3, 99.8, and 99.9 % to Aβ42 stimulation for IL-1β, IL-6, and TNF-α, respectively, Fig. 2b). These data strongly support the anti-inflammatory effects of URMC-099 on Aβ-stimulated microglia.

### Anti-inflammatory effects of URMC-099 in Aβ42-stimulated microglia

**Fig. 3:**
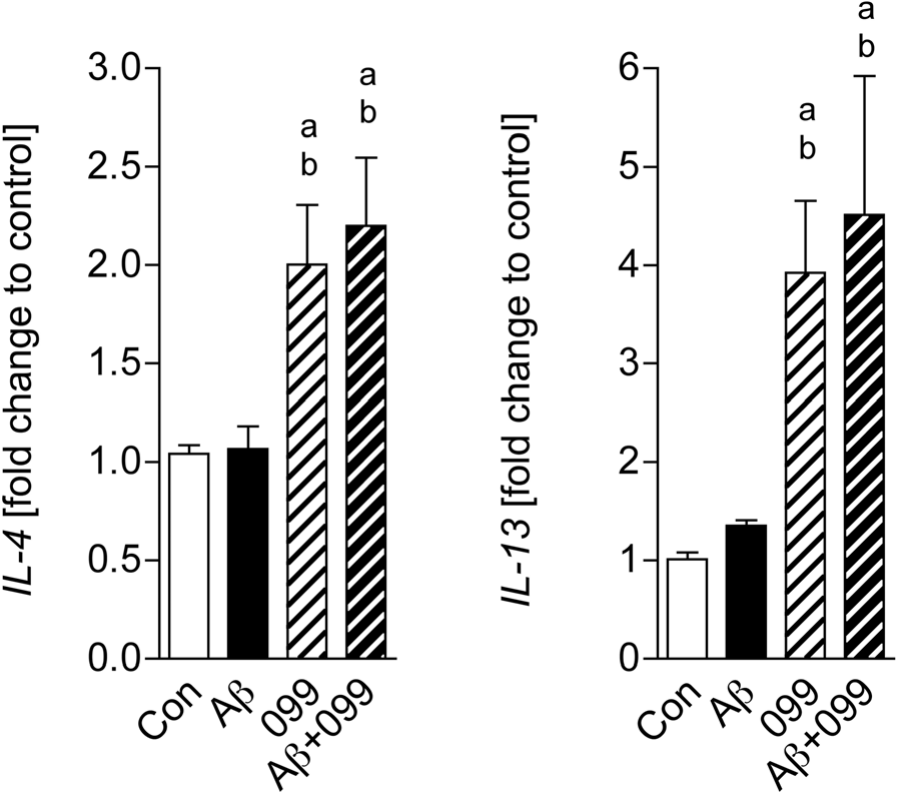
URMC-099 induces gene expression of anti-inflammatory cytokines in Aβ42-stimulated microglia. A conventional RT2-qPCR was performed to measure *IL-4 and IL-13* expression using primer sets (Table 1) and synthesized cDNA with total RNA isolated from murine microglia (*n* = 3 per group). Data are presented as mean ± SEM, ^a,b^ *p* < 0.05, ^a^vs control, ^b^vs Aβ, one-way ANOVA, Newman–Keuls post hoc test

To determine the neuroinflammatory phenotype in URMC-099-treated microglia, total RNA was isolated and RT2-qPCR was performed for genes specific to IL-4 and IL-13. Data were shown as fold change compared to control (Fig. 3). While 30-min Aβ42 stimulation did not alter gene expression of IL-4 and IL-13, URMC-099 significantly induced this expression pattern (increases of 111.1 and 345.7 % in IL-4 and IL-13, respectively, Fig. 3), further supporting the anti-inflammatory effects of URMC-099 on Aβ-stimulated microglia.

### URMC-099 facilitates microglial Aβ uptake

**Fig. 4:**
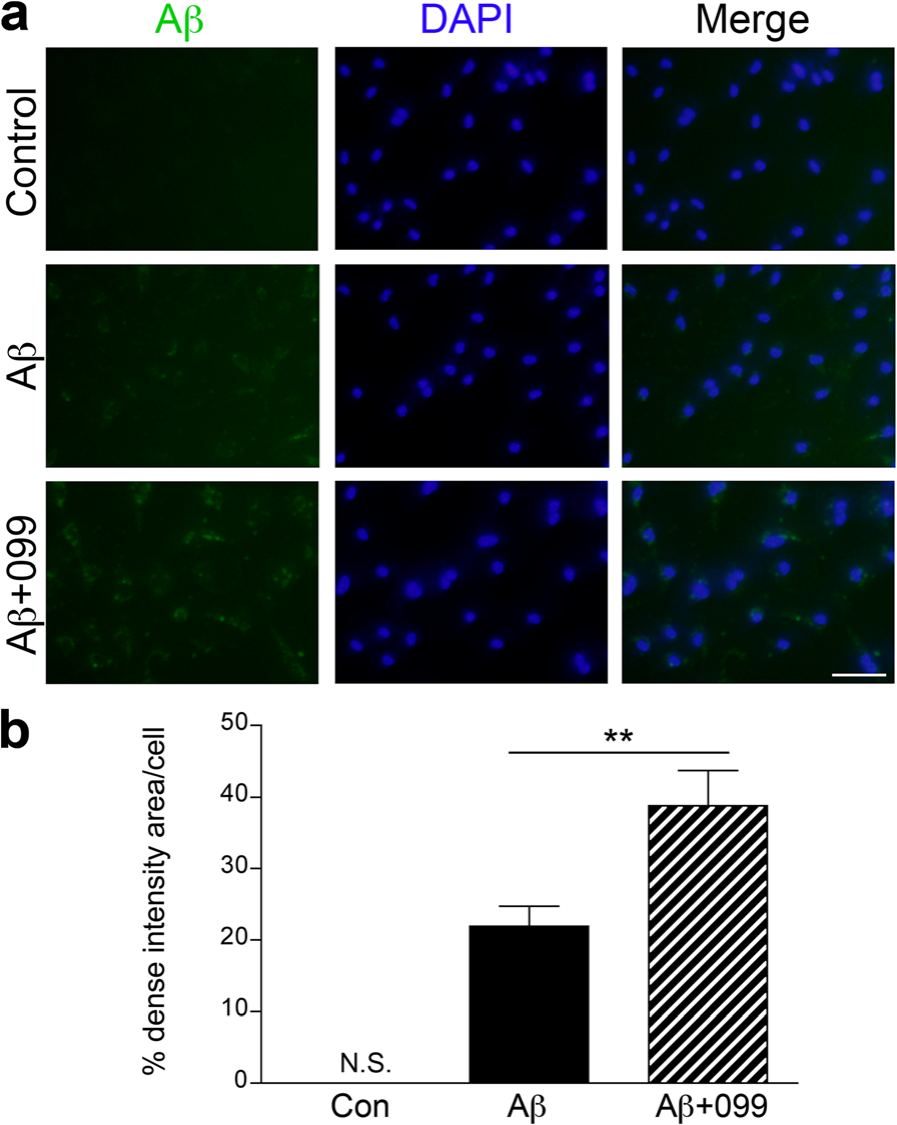
URMC-099 facilitates microglial Aβ-uptake. **a** Primary mouse microglia were incubated with soluble Aβ42 for 30 min, followed by immunofluorescence with anti-Aβ Ab (*green*) and DAPI (*blue*) for nuclear staining (*blue*). Merged captured images were shown. Scale bar, 100 μm. **b** Dense intensity of Aβ42 fluorescence was measured using ImageJ. *Bars* represent mean ± SEM. **p < 0.01 by Student’s *t* test

Since URMC-099 has anti-inflammatory effects in Aβ-stimulated microglia, we hypothesized that co-temporaneous administration of URMC-099 to microglia could accelerate Aβ phagocytosis and subsequent degradation [41–44]. To this end, we tested if URMC-099 could facilitate Aβ42 internalization. URMC-099-treated microglia were incubated with Aβ42 for 30 min, then immunostained using pan-Aβ primary and Alexa 488-secondary antibodies (Fig. 4a). Quantification of immunofluorescent intensity showed that relative immunofluorescence was increased in URMC-099-treated microglia, as compared to Aβ42 treatment only (76.9 % increase, Fig. 4b), suggesting that URMC-099 promotes Aβ phagocytosis.

### URMC-099 alters scavenger receptor expression in microglia

**Fig. 5:**
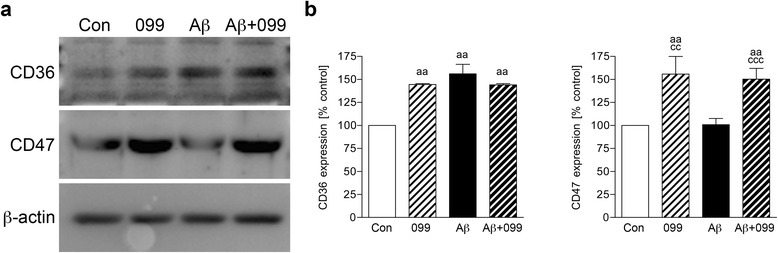
URMC-099 alters scavenger receptor expression in microglia. **a** Representative images of immunoblots for CD36 and CD47 in mouse microglia after a 30-min Aβ42 stimulation. **b** Quantification CD36 and CD47 expression. *Bars* represent mean ± SEM. ^aa,cc^ *p* < 0.01, ^ccc^ *p* < 0.001, ^a^vs control, ^c^vs Aβ, one-way ANOVA, Newman–Keuls post hoc test

A previous study demonstrated that microglial phagocytic capacity is linked to scavenger receptor (SR) expression levels in microglia in AD mouse models [45]. To pursue the mechanism of how URMC-099 facilitates Aβ phagocytosis, we examined the expression of SRs. CD36 and CD47 expression was assessed by immunoblotting (Fig. 5a). CD36 expression was increased in all treated groups (increases of 44.4, 56.1, and 44.1 % in URMC-099 only, Aβ only, and Aβ plus URMC-099 to control, respectively, Fig. 5b), while CD47 expression was significantly increased with URMC-099 treatment (increases of 55.9 and 50.2 % in URMC-099 only and Aβ plus URMC-099 to control, respectively, Fig. 5b), demonstrating URMC-099-specific alteration in SR expression.

### URMC-099 increases Aβ co-localization with Rab7 and Lamp1

**Fig. 6:**
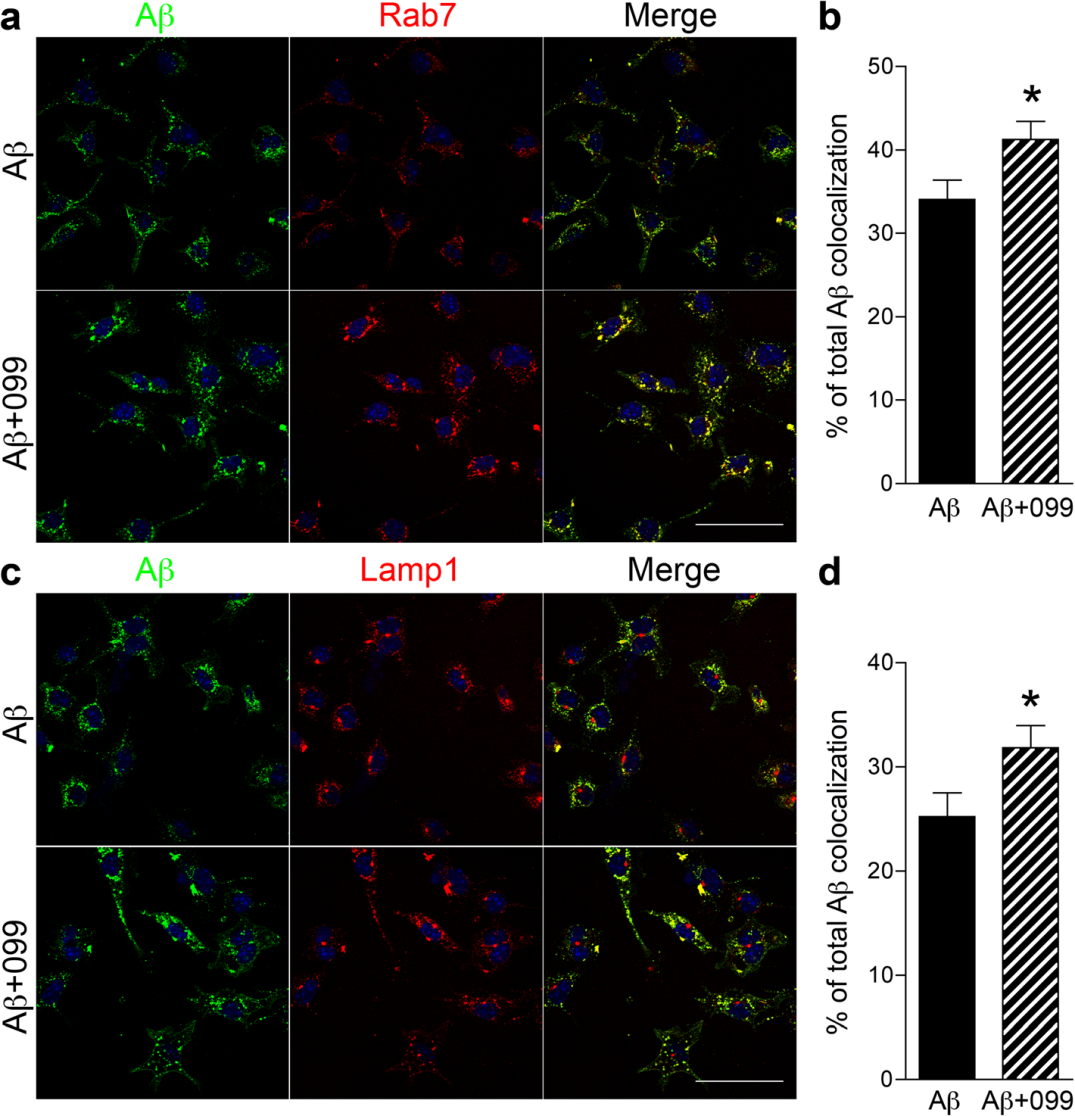
URMC-099 facilitates subcellular co-localization of Aβ42 with Rab7 and Lamp1 in murine microglia. Confocal microscopy shows cellular localization of Rab7 late endosomal component (**a**, *red*) or Lamp1 lysosomal compartment (**b**, *red*) and Aβ42 (*green*). Merged images of Rab7 and Lamp1 are shown. Aβ42 co-localization with Rab7 (**c**) or Lamp1 (**d**) was quantified by ImageJ with a co-localization plugin. Scale bar = 50 μm. *Bars* represent mean ± SEM. *p < 0.05 by Student’s *t* test

**Fig. 7:**
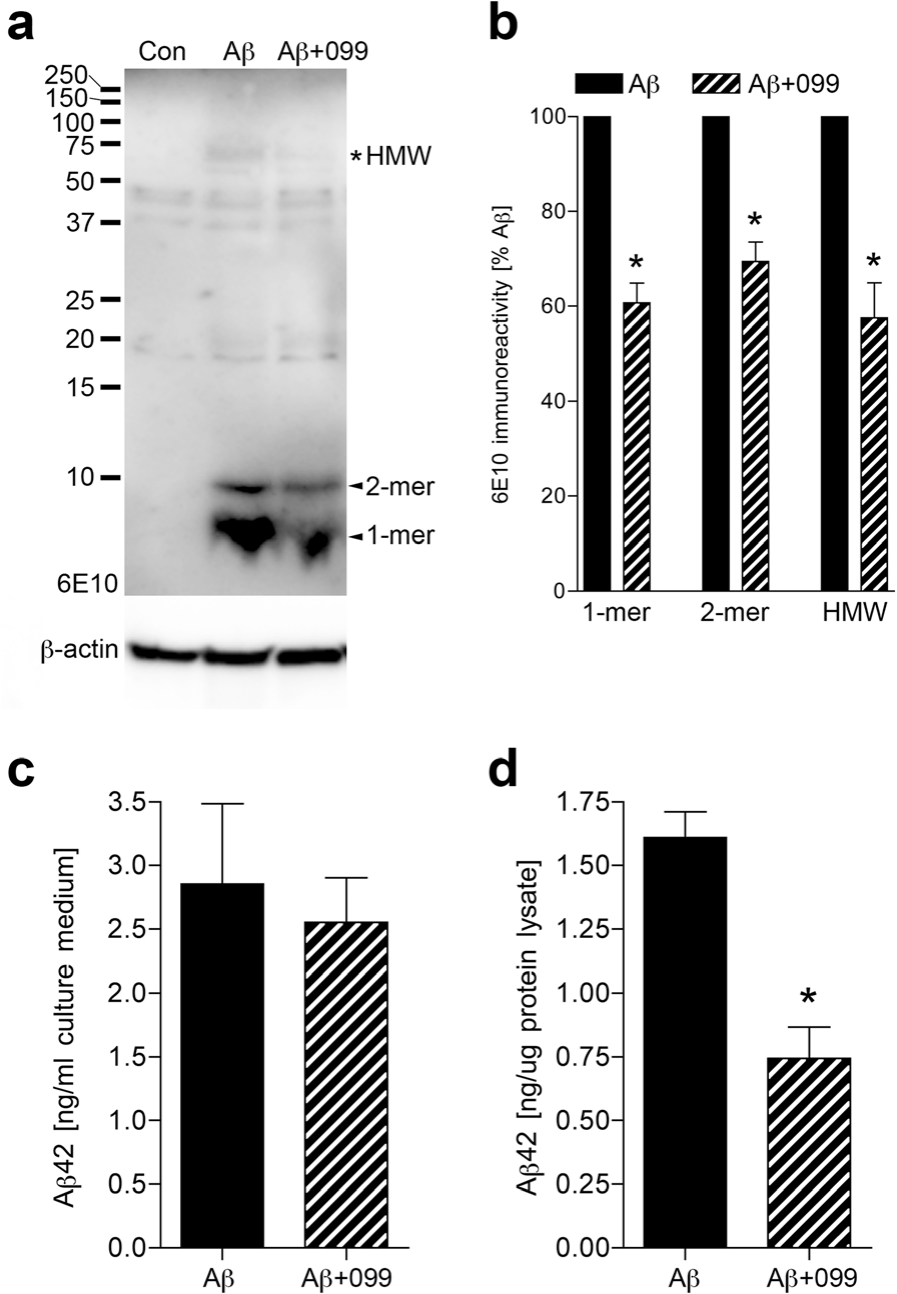
URMC-099 facilitates Aβ42 degradation in murine microglia. **a** Microglia were exposed to Aβ42 for 30 min, followed by wash and culture in fresh media for additional 1 h, and harvested for immunoblot using a 10 % SDS-polyacrylamide Tris-Tricine gel and 6E10 antibody. 1-mer, monomeric Aβ42. 2-mer, dimeric Aβ42. *Asterisk* indicates high molecular weight (HMW) Aβ42. **b** Band luminescent intensities for monomeric, dimeric, and HMW Aβ42 were quantified by ImageJ software. The amounts of Aβ42 in culture media (**c**) and microglial cell lysates (**d**) were measured by human Aβ42-specific ELISA. *Bars* represent mean ± SEM. (*n* = 3 per group). *p < 0.05 vs Aβ, as determined by Student’s *t* test

In response to Aβ binding to SRs, microglia start to engulf Aβ by phagocytosis, and then Aβ enters into the endolysosomal pathway. Thus, we investigated how URMC-099 affects the endolysosomal trafficking underlying Aβ phagocytosis. Microglia treated as described above were immunostained with antibodies to Rab7 (for late endosomes, Fig. 6a) and Lamp1 (for lysosomes, Fig. 6c) at 1-h post-incubation with Aβ. Confocal microscopy demonstrated that co-localization of Rab7 and Lamp1 with Aβ42 was increased in URMC-099-treated microglia, compared to untreated microglia (21.2 and 26.3 % increases in Rab7 and Lamp1, respectively, Fig. 6c, d). To investigate Aβ metabolism, microglia were exposed to Aβ42 for 30 min, washed and cultured in fresh media for additional 1 h, then harvested for immunoblotting (Fig. 7a). Co-treatment with URMC-099 significantly reduced immunoreactivity of monomeric, dimeric, and high molecular weight (HMW) Aβ42, as compared to Aβ42 treatment only (39.3, 30.6, and 42.3 % decreases in monomeric, dimeric, and HMW Aβ42, respectively, Fig. 7b). To validate these results, microglia were exposed to Aβ42 for 30 min, followed by wash and culture in fresh media for additional 1 h, and then Aβ42 release and retention in microglia were quantified using ELISA. While URMC-099 had no effect on Aβ42 release (Fig. 7c), co-treatment with URMC-099 significantly reduced Aβ42 with a 50.2 % reduction (Fig. 7d). These data suggest that URMC-099 promotes microglial endolysosome-mediated degradation.

## Discussion

Microglia are the resident immune cells that serve as the first line of immune defense against invading pathogens in the CNS. The immune response is accelerated by the cell’s abilities to recognize pathogen-associated microbial patterns, induce key co-stimulatory molecules, and secrete cytokines that facilitate induction of the adaptive immune response in disease [46–48]. A principal microglial function is to act as scavenger cells to first phagocytose then clear substances such as Aβ [48–50]. Notably, Aβ is considered to be the initiating factor in AD inducing neuroinflammation, subsequent synaptic and axonal injuries, tau hyperphosphorylation, and ultimately neuronal death [15–17, 51]. With these in mind, a number of therapeutic strategies have been developed for facilitating Aβ removal with the goal of improving disease outcomes. Indeed, anti-inflammatory drugs, amyloid degradation enzymes, and active/passive immunizations have been evaluated as treatment approaches [52–55]. In particular, NSAIDs and antioxidants are effective not only in attenuating the neurotoxins secreted by Aβ-stimulated microglia but also in facilitating microglial phagocytic activity for Aβ clearance in animal models of human disease [1, 25, 26]. To date, there has been no clear validation of any of these approaches for disease-modifying outcomes in patients with AD, thus further studies for alternative disease-modifying strategies are warranted. Hence, we investigated whether URMC-099 could ameliorate Aβ-mediated microglial activation.

URMC-099 was discovered as a result of a large screening for MLK3 inhibitors structurally containing a pyrrolopyridine scaffold with an aryl piperazine side chain [28, 29]; it can modify the production of pro-inflammatory mediators by inhibiting MAPK signaling cascades [36–38]. MLKs are MAPK kinase kinases (MAPKKKs) that function as bonafide serine/threonine kinases, although their catalytic domains have features of both serine/threonine and tyrosine kinases. MLKs regulate the p38/JNK signaling cascades that coordinate and orchestrate numerous immune processes [40]. URMC-099 is an inhibitor specific to MLK3 [28, 29] that is one of the most widely expressed members of the MLK family [56]. MLK3 is expressed in immune effector cells including microglia in the CNS and is activated by cellular and metabolic stress [28, 57–60]. Based on these findings, we posit that URMC-099 is a potential candidate for AD therapeutics. Although it was previously unclear whether Aβ induces classical microglial activation through MLK-MAPK pathways, we showed that URMC-099 reduces phosphorylation of MKK3/MKK4 and p38/JNK and partially protected Aβ-mediated microglial cytotoxicity, suggesting its therapeutic effect targeting MLK-MKK-MAPK pathways in Aβ-mediated neuroinflammation in microglia.

In AD, microglia contribute to tissue injury through eliciting neuroinflammation and changing the CNS microenvironment. Like peripheral macrophages, microglia can be polarized into M1 and M2 phenotypes based on their functional properties [25, 26, 61–63]. Aβ activation of microglia is associated with production of pro-inflammatory cytokines (IL-1β/IL-6/TNF-α), traits associated closely with the M1 phenotype [7, 8, 11, 25, 47, 64]. Alternatively, polarization to an M2 phenotype can initiate an anti-inflammatory and repair phase [1, 25, 26]. The normal homeostatic balance between M1 and M2 phenotypes seems to be disturbed during disease progression in AD with the presence of more M1 microglia appearing coincident with aging and disease [26]. Herein, URMC-099 treatment was shown to inhibit IL-1β/IL-6/TNF-α expression and coordinate with the up-regulation of IL-4 and IL-13 genes. These data suggest that URMC-099 treatment may initiate phenotypic changes by inducing anti-inflammatory cytokines, thus has neuroprotective activities [42–44].

Additionally, microglia in the M2 phase speed Aβ phagocytosis and degradation without neurotoxin production [41–44]. An increase in phagocytosis of Aβ was observed with URMC-099 treatment. Microglial phagocytic capacity is linked to SR expression levels [45]. CD36 is one of the SRs for Aβ and regulates Aβ clearance as well as brain inflammation [65]. While Aβ exposure increased CD36 expression similar to a previous study with BV-2 microglia [66], co-administration of URMC-099 with Aβ failed to reverse this up-regulation. In contrast, URMC-099 increases CD47 expression in microglia both alone and with Aβ. Since CD47 is an integrin-associated transmembrane protein and participates in Aβ uptake and microglial pro-inflammatory responses by forming the receptor complex CD36/CD47/α6β1 integrin, which in turn stimulates microglial phagocytic activity [67–69]. By increasing CD47 activity, our results suggest that URMC-099 facilitates CD36/CD47/α6β1-integrin-mediated microglial phagocytic activity of Aβ. Moreover, URMC-099 facilitates co-localization of Rab7 and Lamp1 with Aβ42, which may promote endolysosomal-mediated Aβ degradation and metabolism. Our data in aggregate suggests that these pathologic events can be altered with the challenge of how and when to optimally use disease-modifying agents such as URMC-099 to restore homeostasis to the brain’s microenvironment.

## Conclusions

URMC-099 inhibits Aβ-mediated phosphorylation of MKK3/4-p38/JNK and pro-inflammatory responses, and up-regulates phagolysosomal trafficking and degradation of Aβ. Such inhibition of phosphorylation by URMC-099 correlates with inhibition of microglial activation. URMC-099 serves as an immune modulatory and neuroprotective agent that may be developed to combat AD.

## Abbreviations

Ab, antibody; AD, Alzheimer’s disease; ANOVA, analysis of variance; Aβ, amyloid-β; BCA, bicinchoninic acid assay; CD, cluster of differentiation; cDNA, complementary deoxyribonucleic acid; CNS, central nervous system; DAPI, diamino-2-phenyl-indol; ELISA, enzyme-linked immunosorbent assay; Gapdh, glyceraldehyde-3-phosphate dehydrogenase; HRP, horseradish peroxidase; IgG, immunoglobulin G; IL, interleukin; JNK, c-Jun amino-terminal kinase; Lamp1, lysosomal-associated membrane protein 1; MAPK, mitogen-activated protein kinase; MCSF, monocyte colony-stimulating factor; MKK, mitogen-activated protein kinase kinase; MLK, mixed-lineage kinase; NSAID, nonsteroidal anti-inflammatory drug; PCR, polymerase chain reaction; PFA, paraformaldehyde; RIPA, radioimmunoprecipitation assay buffer; RNA, ribonucleic acid; SR, scavenger receptor; TBST, Tris-buffered saline containing 0.1 % Tween-20; TNF-α, tumor necrosis factor-α

## Additional files

Additional file 1: Figure S1. URMC-099 treatment for 3, 24, or 48 h has no effect on microglial viability by using the 3-(4,5-dimethylthiazol-2-yl)-2,5-diphenyltetrazolium bromide assay. Data are presented as mean ± SEM. (TIF 1588 kb) Additional file 2: Figure S2. MTT assay of microglia treated with Aβ42 and URMC-099. Data are presented as mean ± SEM. (TIF 613 kb)
