# Supplementary figures and images for: The mixed-lineage kinase 3 inhibitor URMC-099 facilitates microglial amyloid-β degradation
Source: J Neuroinflammation. 2016 Jul 11;13:184. doi: 10.1186/s12974-016-0646-z (PMC4940949; doi:10.1186/s12974-016-0646-z)

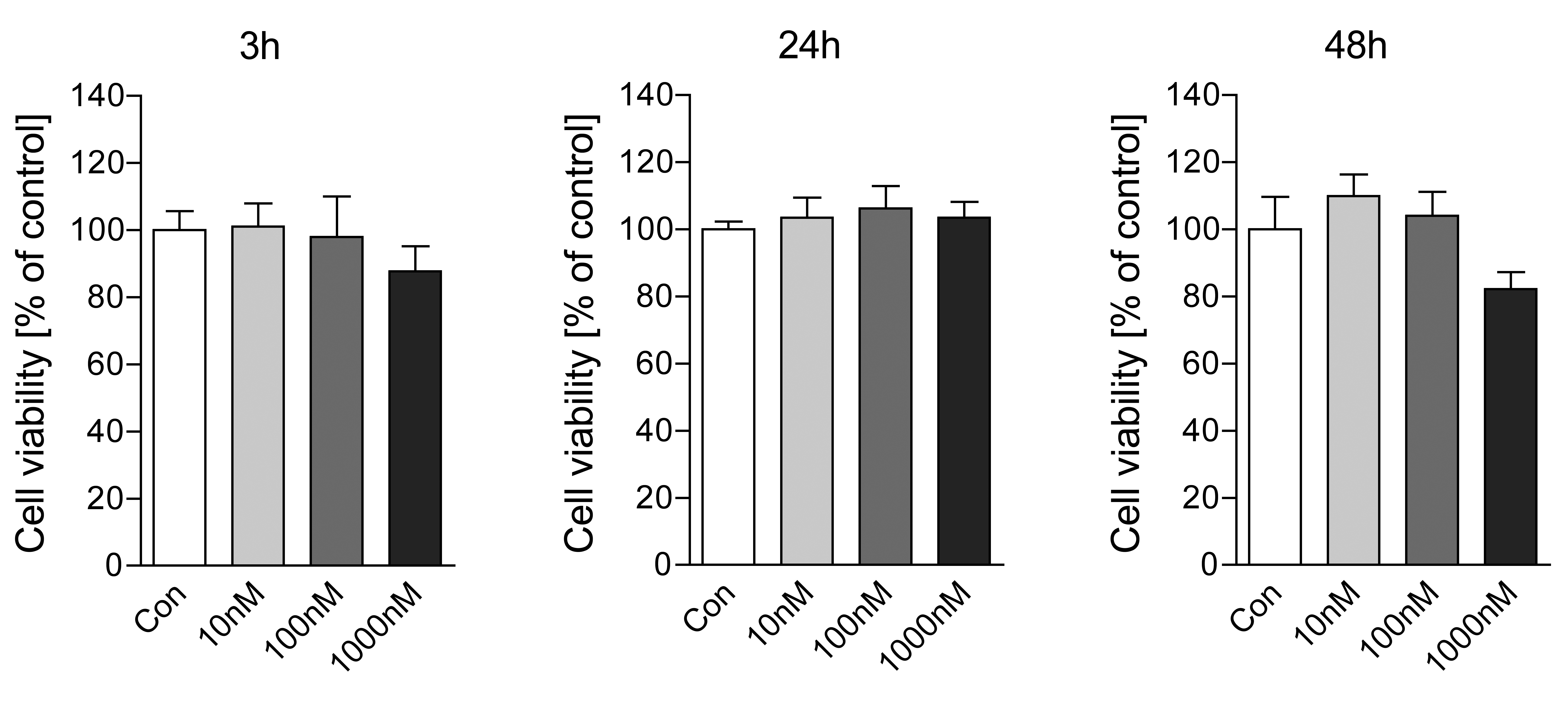

Supplement: Additional file 1: Figure S1. — URMC-099 treatment for 3, 24, or 48 h has no effect on microglial viability by using the 3-(4,5-dimethylthiazol-2-yl)-2,5-diphenyltetrazolium bromide assay. Data are presented as mean ± SEM. (TIF 1588 kb) [file 12974_2016_646_MOESM1_ESM.tif]

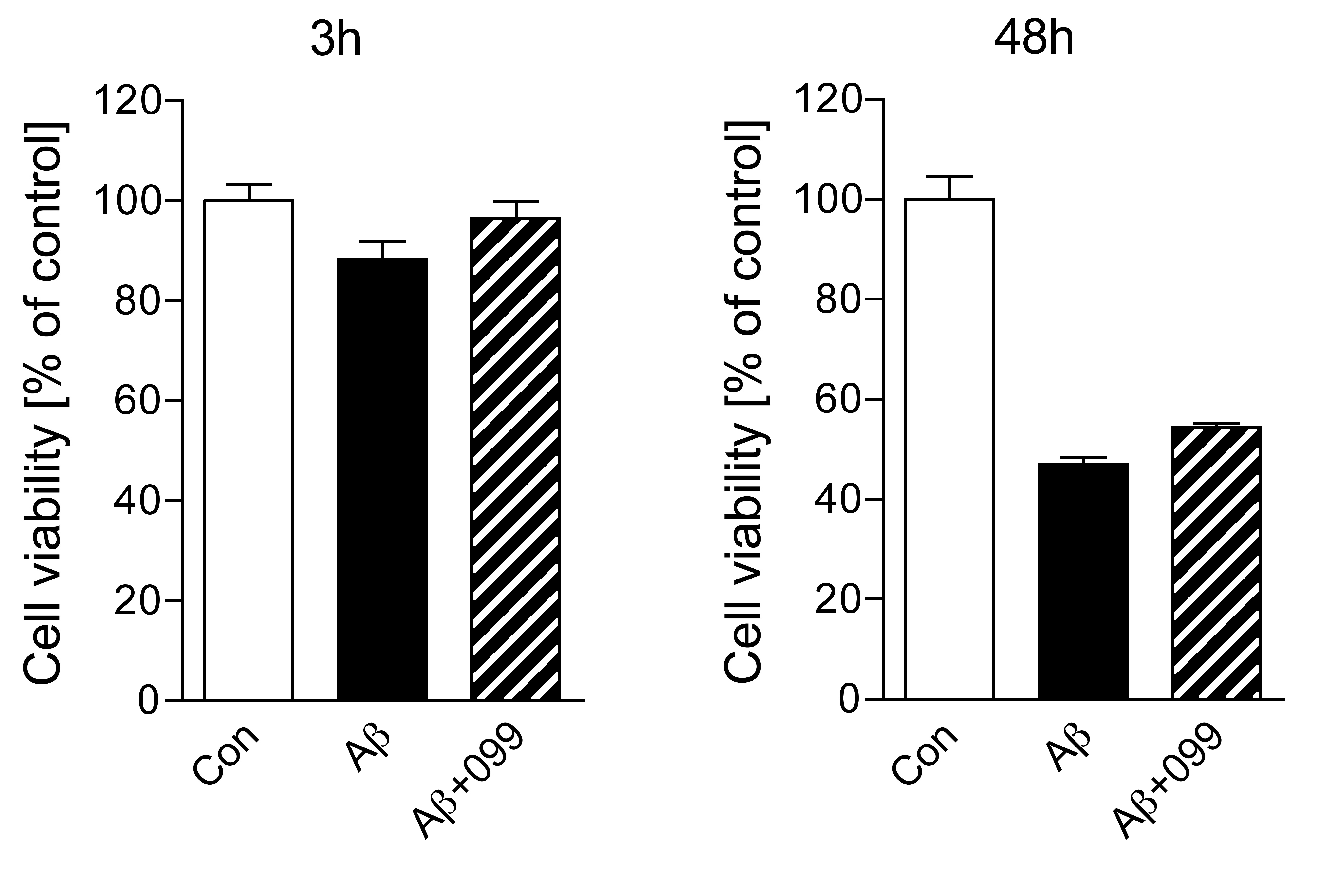

Supplement: Additional file 2: Figure S2. — MTT assay of microglia treated with Aβ42 and URMC-099. Data are presented as mean ± SEM. (TIF 613 kb) [file 12974_2016_646_MOESM2_ESM.tif]
